# Efficacy of resveratrol for the treatment in patients with ulcerative colitis: Study Protocol

**DOI:** 10.1097/MD.0000000000017938

**Published:** 2019-11-15

**Authors:** Yan-hui Chen, Yi Xiang

**Affiliations:** a Department of General Surgery, The First People's Hospital of Xianyang; b Department of Gastroenterology, The Second Affiliated Hospital of Shaanxi University of Chinese Medicine, Xianyang, China.

**Keywords:** efficacy, resveratrol, safety, ulcerative colitis

## Abstract

**Background:**

This study will aim to systematically explore the efficacy of resveratrol for the treatment of patients with ulcerative colitis (UC).

**Methods:**

We will search the electronic databases of MEDLINE, EMBASE, Cochrane Library, Web of Science, Chinese Biomedical Literature Database, and China National Knowledge Infrastructure up to the September 1, 2019 for randomized controlled trials (RCTs) that report on UC who have undergone resveratrol compared with other interventions. All electronic databases will be searched without restrictions of language. Two authors will independently conduct study screen, data extraction, and risk of bias assessment. Any disagreements between 2 authors will be resolved with a third author by discussion or consultation if it is necessary. RevMan 5.3 software will be applied for statistical analysis.

**Results:**

Outcomes include clinical remission, improvement of clinical symptoms, maintenance of remission, relapse rate, endoscopic assessment, histological assessment, quality of life, and adverse events.

**Conclusion:**

This study will provide most recent evidence of resveratrol for the treatment of patients with UC.

**PROSPERO registration number:**

PROSPERO CRD42019150849.

## 1 Introducton

Ulcerative colitis (UC) is a chronic, idiopathic inflammatory disease.^[1,2]^ It is characterized by relapsing and remitting mucosal inflammation.^[3–5]^ It often manifests as diarrhea, rectal bleeding, urgent need to defecate, and abdominal pain.^[6–9]^ It has reported that the incidence steadily increased around the world during the past 2 decades.^[10,11]^ Although its precise etiology is still unknown, several treatments are reported to manage this condition, including nonsteroidal anti-inflammatory drug, antibiotics, anti inflammatory drug, steroid, analgesic, dietary supplement, surgery, supportive care, acupuncture, and moxibusion.^[12–21]^ Of those, resveratrol has been reported to treat UC effectively.^[22–24]^ However, no systematic review has been conducted to assess its efficacy and safety for the treatment of UC. Thus, this study will comprehensively and systematically investigate the efficacy and safety of resveratrol for the treatment of patients with UC.

## 2 Methods

### 2.1 Eligibility criteria for the selection of studies

#### 2.1.1 Type of studies

We will only consider randomized controlled trials (RCTs) focusing on the efficacy and safety of resveratrol for the treatment of patients with UC. However, non-RCTs and quasi-RCTs will be excluded.

#### 2.1.2 Type of participants

We will include patients who were diagnosed as UC without limitations of race, sex, and age.

#### 2.1.3 Type of interventions

All patients in the experimental group must undergo resveratrol alone.

However, all patients in the control group can receive any interventions, but not any single or combined forms of resveratrol.

#### 2.1.4 Type of outcomes

In this study, we will assess the outcomes of clinical remission, improvement of clinical symptoms, maintenance of remission, relapse rate, endoscopic assessment, histological assessment, quality of life, and adverse events.

### 2.2 Search strategy

We will search MEDLINE, EMBASE, Cochrane Library, Web of Science, Chinese Biomedical Literature Database, and China National Knowledge Infrastructure up to the September 1, 2019 without language limitations. We will use the following search terms: ulcerative colitis, colitis ulcerosa, inflammatory bowel disease, resveratrol, randomized controlled trial, clinical study, controlled study, clinical trial, random, and blind. We will build a search strategy sample for MEDLINE in Table 1. We will also adapt similar search strategy to other electronic databases. In addition, we will also search the grey literatures, including dissertations, clinical registry, and reference lists of relevant reviews.

**Table 1:**
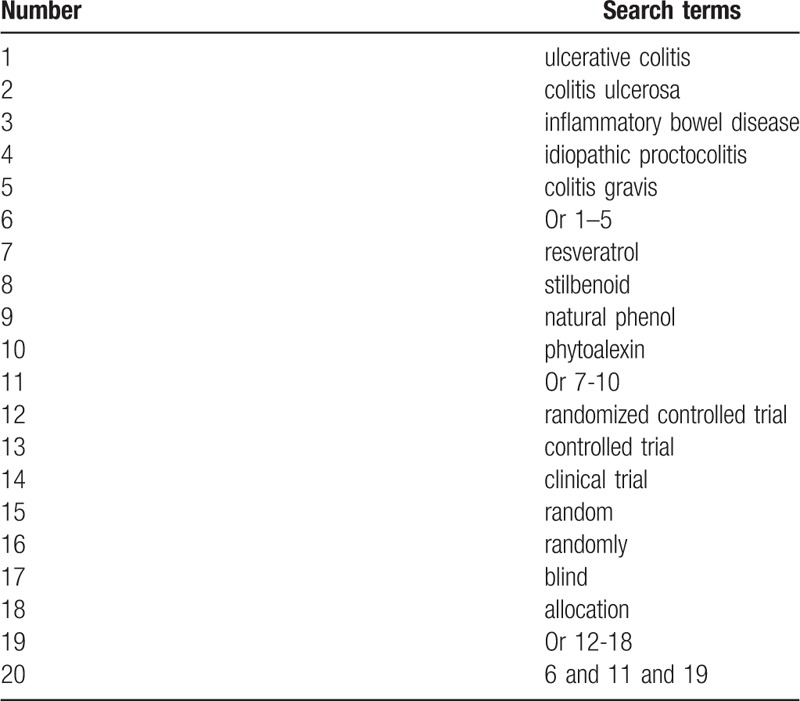
Search strategy for MEDLINE.

### 2.3 Data selection and extraction

#### 2.3.1 Study selection

Two authors will scan the titles and abstracts of all searched studies to identify studies for inclusion initially, and all irrelevant studies will be excluded. At the second stage, we will assess the full texts of remaining studies for eligibility. Any different opinions of study selection between 2 authors will be solved by a third author through discussion. We will exert the process of study selection in the flowchart.

#### 2.3.2 Data extraction

Two authors will independently collect data using piloted data extraction sheet. We will extract study characteristics (title, first author, time of publication, et al), patient characteristics (race, sex, age, inclusion and exclusion criteria, diagnostic criteria, sample size, et al), study setting, study methods (methods of randomization, blind and allocation details, et al), treatment details (intervention name, dosage, frequency, duration, et al), outcomes (all outcome measurements, safety, et al), and funding information. If there are any divergences between 2 authors, we will invite another author to solve them via discussion where required. We will contact primary study authors for any insufficient or missing data if it occurs during the period of data extraction.

### 2.4 Assessment of risk of bias

Two authors will assess the risk of bias for all eligible studies using Cochrane Risk of Bias Tool. This tool has 7 aspects, and we will further judge each aspect as low, unclear and high risk of bias. A third author will help to solve any disagreements between 2 authors by discussion or consultation if necessary.

### 2.5 Data synthesis and analysis

We will use RevMan 5.3 software for statistical analysis in this study. We will express continuous outcome data as mean difference or standardized mean difference with 95% confidence intervals (CIs), and dichotomous data as risk ratio with 95% CIs. We will evaluate heterogeneity among included studies using *I*^2^ test. *I*^2^ ≤ 50 means low heterogeneity, and a fixed-effects model will be applied. *I*^2^ > 50% exerts significant heterogeneity, and a random-effects model will be utilized. When the heterogeneity is low, we will perform meta-analysis with low heterogeneity if more than 2 studies using the same treatments, comparators, and outcomes. When the heterogeneity is substantial, we will carry out subgroup analysis and meta-regression to explore possible factors that may cause significant heterogeneity.

### 2.6 Subgroup analysis

We will conduct subgroup analysis based on the different study quality, treatments, and outcome measurements.

### 2.7 Sensitivity analysis

If sufficient data are available, we will plan for sensitivity analysis to check the robustness of pooled results by removing studies with high risk of bias.

### 2.8 Reporting bias

If sufficient studies are included, we will perform Funnel plot^[25]^ and Egger regression test^[26]^ to check the reporting bias among the eligible studies.

### 2.9 Ethics and dissemination

This study will not require ethic approval, because no individual patient data will be used. We are expected to publish this study at a peer-reviewed journal.

## 3 Discussion

Previous studies have found that resveratrol can effectively treat patients with UC. However, its results are still inconsistent and inconclusive. Thus, this study will systematically assess the efficacy and safety of resveratrol for the treatment of patients with UC. We hope this study may provide convincing evidence to the clinical practice and health related policy maker.

This study still has several potential limitations. First, different study quality may affect the heterogeneity among studies. Second, different doses of resveratrol may also cause clinical heterogeneity.

## Author contributions

**Conceptualization:** Yan-hui Chen, Yi Xiang.

**Data curation:** Yan-hui Chen, Yi Xiang.

**Formal analysis:** Yan-hui Chen, Yi Xiang.

**Investigation:** Yan-hui Chen.

**Methodology:** Yi Xiang.

**Project administration:** Yan-hui Chen.

**Resources:** Yi Xiang.

**Software:** Yi Xiang.

**Supervision:** Yan-hui Chen.

**Validation:** Yan-hui Chen, Yi Xiang.

**Visualization:** Yan-hui Chen, Yi Xiang.

**Writing – original draft:** Yan-hui Chen, Yi Xiang.

**Writing – review & editing:** Yan-hui Chen, Yi Xiang.
